# Predictive model for the preoperative assessment and prognostic modeling of lymph node metastasis in endometrial cancer

**DOI:** 10.1038/s41598-022-23252-3

**Published:** 2022-11-08

**Authors:** Yuka Asami, Kengo Hiranuma, Daisuke Takayanagi, Maiko Matsuda, Yoko Shimada, Mayumi Kobayashi Kato, Ikumi Kuno, Naoya Murakami, Masaaki Komatsu, Ryuji Hamamoto, Takashi Kohno, Akihiko Sekizawa, Koji Matsumoto, Tomoyasu Kato, Hiroshi Yoshida, Kouya Shiraishi

**Affiliations:** 1 Division of Genome Biology, National Cancer Center Research Institute, 5-1-1 Tsukiji, Chuo-Ku, Tokyo, 104-0045 Japan; 2 Department of Obstetrics and Gynecology, Showa University School of Medicine, Tokyo, 142-8666 Japan; 3 Department of Obstetrics and Gynecology, Faculty of Medicine, Juntendo University, Tokyo, 113-8421 Japan; 4 Department of Gynecology, National Cancer Center Hospital, Tokyo, 104-0045 Japan; 5 Department of Radiation Oncology, National Cancer Center Hospital, Tokyo, 104-0045 Japan; 6 Division of Medical AI Research and Development, National Cancer Center Research Institute, Tokyo, 104-0045 Japan; 7 Cancer Translational Research Team, RIKEN Center for Advanced Intelligence Project, Tokyo, 103-0027 Japan; 8 Division of Diagnostic Pathology, National Cancer Center Hospital, 5-1-1 Tsukiji, Chuo-Ku, Tokyo, 104-0045 Japan

**Keywords:** Endometrial cancer, Tumour biomarkers

## Abstract

Lymph node metastasis (LNM) is a well-established prognostic factor in endometrial cancer (EC). We aimed to construct a model that predicts LNM and prognosis using preoperative factors such as myometrial invasion (MI), enlarged lymph nodes (LNs), histological grade determined by endometrial biopsy, and serum cancer antigen 125 (CA125) level using two independent cohorts consisting of 254 EC patients. The area under the receiver operating characteristic curve (AUC) of the constructed model was 0.80 regardless of the machine learning techniques. Enlarged LNs and higher serum CA125 levels were more significant in patients with low-grade EC (LGEC) and LNM than in patients without LNM, whereas deep MI and higher CA125 levels were more significant in patients with high-grade EC (HGEC) and LNM than in patients without LNM. The predictive performance of LNM in the HGEC group was higher than that in the LGEC group (AUC = 0.84 and 0.75, respectively). Patients in the group without postoperative pathological LNM and positive LNM prediction had significantly worse relapse-free and overall survival than patients with negative LNM prediction (log-rank test, *P* < 0.01). This study showed that preoperative clinicopathological factors can predict LNM with high precision and detect patients with poor prognoses. Furthermore, clinicopathological factors associated with LNM were different between HGEC and LGEC patients.

## Introduction

Endometrial cancer (EC), the fifth most frequent cancer in women, accounts for an estimated 382,000 new cancer cases worldwide and 90,000 deaths annually^1^. In 2018, 17,089 patients were newly diagnosed with EC and 2597 patients with EC died in Japan^2^. In the United States, the mortality rate due to EC, which decreased initially, is on the rise since 2010, and nearly 12,550 deaths have been estimated in 2022^3^. EC is the most commonly observed gynecological malignancy^4^ and is classified as type 1, grade 1, or grade 2 endometrioid carcinoma, which is regarded as low-grade EC (LGEC) and accounts for approximately 80% of cases; type 2, which has a high histologic grade and aggressive clinical behavior, accounts for the remaining 20%^5,6^. High-grade endometrial carcinomas (HGECs) are a heterogeneous group of tumors that include grade 3 endometrioid, serous, and clear cell carcinomas. Although the overall prognosis of patients with EC is generally good, with an 80% overall survival (OS) at 5 years, 15–20% of patients with a low-risk profile still experience recurrence^7^. Furthermore, the outcomes for patients with EC with systemic recurrence are poor, with a median survival hardly exceeding 12 months^8^.

In EC, the assessment of lymph node metastasis (LNM) is an important factor in determining treatment strategies and predicting prognosis. The LNM status is closely related to poor prognosis and is an important factor in EC staging and determining the need for adjuvant therapy^9–11^. Two randomized trials have shown no therapeutic benefit of systemic lymphadenectomy in patients with low risk for recurrence^12,13^. It has also been reported that preoperative stratification by imaging and histological assessments permits a reduction in lymphadenectomy to approximately 50%^14^. However, lymphadenectomy should be performed in patients with non-endometrioid histology or deeply infiltrating high-grade disease, both of which are known to have more aggressive behavior, even though lymphadenectomy has a 10–20% risk of lower-extremity lymphedema and a 10–25% risk of lymphocele development^15,16^. Although lymphadenectomy should be performed based on a balance of risks and benefits, an international consensus has not yet been reached on the eligibility criteria for it^17^.

The preoperative or postoperative assessment of LNM often uses histology, serum cancer antigen 125 (CA125) level, myometrial invasion (MI), and/or lymphadenopathy by imaging as a part of the preoperative workup^18,19^. Although there have been several reports of preoperative LNM risk assessment in patients with EC, the current preoperative risk assessment is moderate (sensitivity 67–92%)^18,20–26^. Todo et al. reported that preoperative clinicopathological factors such as volume index as assessed by magnetic resonance imaging (MRI), CA125, histological subtype, and grade according to biopsy are associated with LNM of EC^22^; further, they constructed a scoring system for prediction of LNM. The diagnostic predictive performance of this scoring system was 92% for sensitivity and 53% for specificity^23^. Similarly, Kang et al. reported that high CA125 and three MRI parameters (deep MI, enlarged lymph nodes [LNs], and extrauterine extension) were significantly associated with LNM among patients with a low-risk for recurrence. They defined a predicted probability of less than 4%, and developed criteria (Korean Gynecologic Oncology Group [KGOG] criteria) for identifying patients at low risk for LNM^24^; the criteria were validated in a prospective multicenter observational study. The diagnostic predictive performance of the KGOG criteria was 85% for sensitivity, 56% for specificity, and 70% for the receiver operating characteristic (ROC) curve area^19^. Only a few studies have verified that the prediction of LNM is associated with postoperative prognosis. To develop a highly accurate and precise prediction model using preoperative factors, it is necessary to perform many validation studies and develop new methods.

To provide appropriate treatment to patients with suspected LNM, we assessed whether a prediction model constructed by machine learning algorithms using preoperative clinicopathological factors can be a useful tool for selecting patients who need lymphadenectomy. We also investigated how preoperative pathological factors affect LNM predictions on clinical outcomes.

## Materials and methods

### Two cohort studies consisting of NCCH and SUH

We conducted two retrospective cohort studies. Creasman et al. reported that a minimum of 10 LNs were removed during the surgery for EC. Thus, we adopted 10 LNs as our minimum cutoff^27^. Details on case selection are provided in the Supplementary Methods. All patients who had 10 or more LNs removed in addition to the mainstay surgery (hysterectomy, bilateral oophorectomy) between 2007 and 2018 at the National Cancer Center Central Hospital (NCCH) were included in this study to extract a strictly node-negative group that did not include clinically undetectable LNM. A total of 125 EC patients were enrolled in the NCCH cohort. Patients receiving neoadjuvant chemotherapy were excluded. This study was approved by the Institutional Review Board of the National Cancer Center Research Institute (2017–331).

Next, 129 EC patients who had undergone initial surgery (hysterectomy, bilateral salpingo-oophorectomy, and resection of 10 or more LNs) after being diagnosed between 2006 and 2017 at the Showa University Hospital (SUH) were enrolled in the SUH cohort. After obtaining approval from the institutional ethical and research review boards of SUH (approval number: 2544), this study was conducted following the ethical guidelines of the Declaration of Helsinki.

Table 1 summarizes the patient characteristics and preoperative clinicopathological factors for the 254 cases comprising the NCCH and SUH cohorts. Pathological characteristics of the patients' resected samples are summarized in Supplementary Table S1 for the NCCH cohort and Supplementary Table S2 for the SUH cohort. The general guidelines for the treatment of EC at each institution are described in the Supplementary Method.

**Table 1:** Clinicopathological characterisitics of 254 endometrial cancer patients consisting of the National Cancer Center Hospital and the Showa University Hospital cohorts.

Category	Lymph node metastasis (NCCH cohort)		Lymph node metastasis (SUH cohort)	
	Positive [n = 51] (%)	Negative [n = 74] (%)	Positive [n = 29] (%)	Negative [n = 100] (%)
Age, median (range) [years]	57 (29–89)	61 (32–84)	58 (24–79)	61 (32–78)
**Histological grade by preoperative endometrial biopsy**				
Low-grade endometrial cancer (%)	26 (51.0)	45 (60.8)	21 (72.4)	73 (73.0)
High-grade endometrial cancer (%)	25 (49.0)	29 (39.2)	8 (27.6)	27 (27.0)
**MRI findings**				
Myometrial invasion				
< 50%	14 (27.5)	47 (63.5)	10 (34.5)	68 (68.0)
≥ 50%	37 (72.5)	27 (36.5)	19 (65.5)	32 (32.0)
Enlarged lymph nodes				
Negative (%)	33 (64.7)	67 (90.5)	24 (82.8)	95 (95.0)
Positive (%)	18 (35.3)	7 (9.5)	5 (17.2)	5 (5.0)
Tumor diameter, median (range) [mm]	56 (27–122)	39.5 (0–130)	51 (9–137)	35.5 (0–92)
Serum CA125, median (range) [U/mL]	50 (7–1403)	16 (4–448)	70.3 (13.3–849)	23.2 (5.4–521)

*NCCH* National Cancer Center Hospital, *SUH* Showa University Hospital, *MRI* Magnetic resonance imaging, *CA125* Cancer antigen 125.

In the NCCH cohort, the general requirements for informed consent for the use of their samples in the research were obtained at their first visit to the NCCH. Information obtained in our study using samples collected after obtaining general informed consent from participants has been summarized on the NCCH website. Patients were free to revoke their presumed consent at any time point. We only used samples from patients who did not revoke their consent. Similarly, we informed patients treated before 2000 that the information summary of our study is published on the official NCCH website. Patients who refused to provide consent for the use of their residual samples were excluded from this study. The clinical data used in this study were collected from the patients' medical records. Written informed consent was obtained from all patients in the SUH cohort.

### Preoperative clinicopathological factors

Preoperative endometrial biopsy specimens from the NCCH and SUH cohorts were evaluated by at least two pathologists at each institution. In this study, grades 1 and 2 endometrioid endometrial carcinomas were defined as LGEC, and other histological or unknown grade endometrial carcinomas were defined as HGEC. In both cohorts, MRI was routinely used for the preoperative work-up of the patients with EC. Each patient's MRI data were examined by two radiologists at each institution. MI was defined as less than 50% invasion and 50% or more invasion on the axial and sagittal images, respectively. LNs with their short axes longer than 1 cm were considered to be enlarged. Tumor diameter (TD) was defined as the maximum diameter on the sagittal T2-weighted images. TD measurements were used to obtain the ROC curves for LNM. The ROC curve was used to calculate the cut-off value (TD: 47 mm). Serum CA125 levels were determined by chemiluminescent immunoassay using the preferred assay method of each institution. To determine the relationship between the measured serum CA125 levels and pathologic factors, a population should be divided into premenopausal and postmenopausal groups because serum CA125 levels are affected by ovarian hormones and aging^28–31^. In the current study, the patients were divided into two groups according to their menopausal status. The ROC of LNM was obtained based on the CA125 value and the cutoff value was determined (52.3 U/mL [non-menopausal] and 48 U/mL [menopausal]).

### Data splitting

The NCCH cohort (n = 125) was randomly divided into the NCCH training set comprising 75 patients and the NCCH test set with 50 patients; no significant differences in clinicopathological factors between the two sets were found (Supplementary Table S3). This resulted in the allocation of 33 patients with LNM and 42 patients without LNM to the NCCH training set, and 18 patients with LNM and 32 patients without LNM to the NCCH test set.

### Construction and validation of the prediction models for LNM

In this study, the models were constructed using three methods of logistic regression (LR) classifiers as the baseline, in addition to supervised machine learning classifiers of support vector machines (SVM) and random forests (RF). SVM is a method for determining and classifying discriminative thresholds from a data distribution, while RF is used to classify data by collecting a multitude of decision trees. All the classifiers were implemented using the R package randomForest, kernlab, and glm2 (method “ksvm” for SVM and “randomForest” for RF). Machine learning classifiers were trained using repeated five fold cross-validation of the training dataset. Each prediction model was constructed using the NCCH training data and its predictive performance was validated using the NCCH test data and the SUH cohort.

### Statistical analysis

Statistical analysis was performed using R software ver. 4.1.0 (R Foundation, Vienna, Austria) and JMP version 15.0.0 software (SAS Institute Inc., Cary, NC, USA). Variables that achieved statistical significance in the univariate analysis were subsequently included in the multivariate analysis. The level of statistical significance was set at *P* < 0.05. In logistic regression, we adopted the rule of 10 events per variable for the number of variables included in the multivariate analysis. Therefore, for the multivariate analysis, variables with the highest odds ratios in the univariate analysis were selected. Cumulative survival was estimated using the Kaplan–Meier method, and the difference in survival between the two groups was analyzed using the log-rank test. The effects of variables on OS or relapse-free survival (RFS) were determined via univariate and multivariate analyses using the Cox proportional hazard model with R and JMP software.

### Ethical approval

The study protocol was approved by the Institutional Review Board of the National Cancer Center Research Institute and of Showa University (Approval Numbers 2017–331 and 2544, respectively), and the study was conducted following the ethical guidelines of the Helsinki Declaration. Written informed consent was obtained from all the patients using an opt-out form. Patients who refused to provide consent were excluded from the study.

### Informed consent

In the NCCH cohort, the general requirements for informed consent for the use of their samples in the research were obtained at their first visit to the NCCH. Information obtained in our study using samples collected after obtaining general informed consent from participants has been summarized on the NCCH website. Patients were free to revoke their presumed consent at any time point. We only used samples from patients who did not revoke their consent. Similarly, we informed patients treated before 2000 that the information summary of our study is published on the official NCCH website. Patients who refused to provide consent for the use of their residual samples were excluded from this study. The clinical data used in this study were collected from the patients’ medical records. Written informed consent was obtained from all patients in the SUH cohort.

## Results

### Association of preoperative clinicopathological factors with the risk for LNM

The clinical characteristics and pathological data of 254 patients are summarized in Table 1. Deep MI, enlarged LNs, large TD (as determined by MRI), and high serum CA125 levels were significantly higher in patients with than without LNM (*P* < 0.01). In both cohorts, there was no difference in the distribution of biopsy histological subtypes and grades between patients with and without LNM (Table 2). Multivariate analysis revealed that deep MI, enlarged LNs, and high serum CA125 levels were associated with the risk of LNM in the NCCH cohort. Even in the SUH cohort, univariate analysis showed that deep MI, enlarged LNs, large TD, and high serum CA125 levels were higher in patients with than without LNM (*P* < 0.05), and there was no difference in the frequency of biopsy histological types between patients with and without LNM (Table 2B). Multivariate analysis revealed that high serum CA125 levels were associated with the risk of LNM. In the combined analysis of 254 patients from the NCCH and SUH cohorts, deep MI, large TD, enlarged LNs, and high serum CA125 levels were independently associated with LNM in the multivariate analysis (Table 2C).

**Table 2:** Preoperative clinicopathological factors and risk of lymph node metastasis in patients with endometrial cancer.

Category	Univariate			Multivariate		
	OR	(95%CI)	*P* value	OR	(95%CI)	*P* value
**(A) NCCH cohort (n = 125)**						
Biopsy histology (HGEC / LGEC)	1.49	(0.73–3.07)	0.28	–	–	–
MRI						
Myometrial invasion (≥ 50% / < 50%)	4.60	(2.12–10.0)	< 0.01	3.96*	(1.60–9.78)	< 0.01
Tumor diameter (high / low)^1^	4.04	(1.89–8.64)	< 0.01	1.97*	(0.80–4.82)	0.14
Enlarged lymph nodes (positive / negative)	5.22	(1.98–13.7)	< 0.01	4.45*	(1.41–14.0)	0.01
Serum CA125 level (high / low)^2^	8.13	(3.34–19.7)	< 0.01	5.70*	(2.09–15.5)	< 0.01
**(B) SUH cohort (n = 129)**						
Biopsy histology (HGEC / LGEC)	1.03	(0.41–2.60)	0.95	–	–	–
MRI						
Myometrial invasion (≥ 50% / < 50%)	4.04	(1.68–9.67)	< 0.01	2.42*	(0.89–6.61)	0.08
Tumor diameter (high / low)^1^	2.56	(1.09–5.99)	0.03	1.42*	(0.51–3.95)	0.50
Enlarged lymph nodes (positive / negative)	5.22	(1.98–13.7)	0.04	2.26*	(0.42–12.2)	0.34
Serum CA125 level (high / low)^2^	13.4	(5.00–35.9)	< 0.01	9.40*	(3.31–26.7)	< 0.01
**(C) Combined NCCH cohort and SUH cohort (n = 254)**						
Biopsy histology (HGEC / LGEC)	1.48	(0.86–2.56)	0.16	–	–	–
MRI						
Myometrial invasion (≥ 50% / < 50%)	4.55	(2.57–8.06)	< 0.01	3.28**	(1.69–6.36)	< 0.01
Tumor diameter (high / low)^1^	3.29	(1.89–5.74)	< 0.01	1.73**	(0.89–3.37)	0.11
Enlarged lymph nodes (positive / negative)	5.45	(2.55–11.7)	< 0.01	3.59**	(1.40–9.17)	< 0.01
Serum CA125 level (high / low)^2^	8.24	(4.50–15.1)	< 0.01	6.98**	(3.43–14.2)	< 0.01

*OR* Odds ratio, *CI* Confidence interval, *NCCH* National Cancer Center Hospital, *HGEC* High-grade endometrial cancer, *LGEC* Low-gradeendometrial cancer, *MRI* Magnetic resonance imaging, *CA125* Cancer antigen 125, *SUH* Showa University Hospital.

*Adjusted by statistically significant variables in univariate analysis. **Adjusted by myometrial invasion, enlarged lymph nodes, and serum CA125 level. ***Adjusted by cohort and statistically significant variables in univariate analysis.

^1^High level, > 47 mm. ^2^High level, > 52.3 U/mL(unmenopause) , > 48 U/mL(menopause).

### Construction of predictive models for LNM detection using preoperative clinical factors

**Figure 1:**
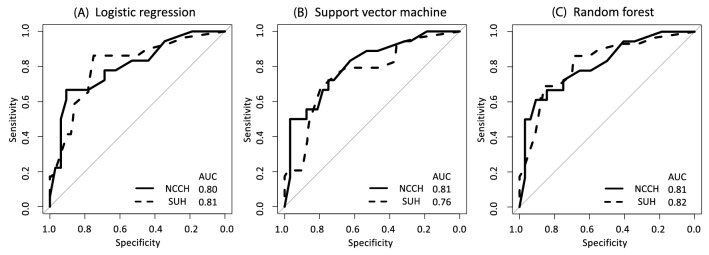
Performance of the preoperative predictive model for lymph node metastasis in endometrial cancer (training set: National Cancer Center Hospital training set (*n* = 75), test set: NCCH test set (*n* = 50), and Showa University Hospital set *(n* = 129). (**A**) Receiver operating characteristic (ROC) curves using logistic regression. (**B**) ROC curves using support vector machine. (**C**) ROC curves using random forest. The solid and dashed lines show the NCCH and the SUH test sets, respectively. *AUC* The area under the ROC curve, *NCCH* National Cancer Center Hospital, *SUH* Showa University Hospital.

We investigated whether a predictive model for LNM could be constructed using the results of routine preoperative examinations, including MI, TD, LNs enlargement, biopsy histology, and serum CA125 levels. Predictive models were constructed for the NCCH training set (n = 75) using three methods: (A) LR, supervised machine learning with (B) SVM, and (C) RF. Models were validated on the test sets of the NCCH (n = 50) and SUH (n = 129) cohorts. The area under the ROC curve (AUC) was calculated to evaluate the predictive power of each model. Nearly all methods showed a high predictive performance above AUC 0.80, which was similar to the results of validation by other cohorts, including the SUH and NCCH test sets (Fig. 1).

### Summary of the previous reported predictive performance

To evaluate the predictive performance of LNM in the present study, we compared it with the previously reported LNM prediction algorithm using preoperative clinical factors. In this study, both the RF and LR using clinical factors showed that our model had slightly lower sensitivity and higher specificity than previously reported models, although the positive likelihood ratio was higher than previously reported (Table 3).

**Table 3:** Comparison of the preoperative prediction models for lymph node metastasis in patients with endometrial cancer.

Study	Cohort	No of patients	No of patients with LNM (%)	Patient selection	Clinical factors	Positive LH (95% CI)	Negative LH (95% CI)	Sensitivity (95% CI)	Specificity (95% CI)	ROC curve area
**(A) Random forest**										
This study	NCCH Training set	75	33 (44%)	Any	Serum CA125, Biopsy histological type, MRI MI, MRI enlarged LNs, MRI TD	4.00 (2.10–7.86)	0.40 (0.27–0.61)	0.67 (0.58–0.76)	0.83 (0.74–0.90)	NA
	NCCH Test set	50	18 (36%)			2.67 (1.38–4.77)	0.44 (0.23–0.80)	0.67 (0.49–0.81)	0.75 (0.65–0.83)	0.81
	SUH Test cohort	129	29 (22.5%)			3.14 (2.02–4.43)	0.40 (0.23–0.63)	0.69 (0.54–0.81)	0.78 (0.74–0.82)	0.82
**(B) Logistic regression**										
This study	NCCH Training set	75	33 (44%)	Any	Serum CA125, Biopsy histological type, MRI MI, MRI enlarged LNs, MRI TD	3.50 (1.91–6.53)	0.41 (0.27–0.64)	0.67 (0.49–0.81)	0.78 (0.68–0.86)	0.84
	NCCH Test set	50	18 (36%)			3.05 (1.52–5.73)	0.43 (0.23–0.76)	0.67 (0.55–0.77)	0.81 (0.71–0.89)	0.80
	SUH Test cohort	129	29 (22.5%)			3.71 (1.98–6.68)	0.60 (0.43–0.80)	0.48 (0.34–0.61)	0.87 (0.83–0.91)	0.81
Todo*Am J Obstet Gynecol (2003)	EC patients treated at 3 hospitals from 1993–2000	214	31 (14.5%)	Any	Serum CA125, Biopsy histological type and grade, MRI MI, MRI tumor volume index	2.25 (1.75–2.53)	0.21 (0.08–0.47)	0.87 (0.72–0.95)	0.61 (0.59–0.63)	NA
Todo*Gynecol Oncol (2007)	EC patients treated at 13 hospitals from 2003–2005	211	36 (17.1%)			1.93 (1.59–2.10)	0.16 (0.05–0.42)	0.92 (0.79–0.97)	0.53 (0.50–0.54)	NA
Kang J Clin Oncol (2012)	Training cohort (EC patients treated at 6 hospitals from 2002–2008)	360	45 (12.5%)	Any	Serum CA125, Biopsy histological type, MRI MI, MRI enlarged LNs, MRI extension beyond corpus	NA	NA	NA	NA	0.85
	Validation cohort (EC patients treated at 6 hospitals from 2002–2008)	180	23 (12.8%)			NA	NA	NA	NA	0.89
Kang Cancer (2017)	Prospective, multicenter cohort study from 20 hospitals in 3 countries (from 2012–2014)	529	53 (10.0%)	Low-risk**	Serum CA125, Biopsy histological type, MRI MI, MRI enlarged LNs MRI extrauterine spread	1.91 (1.61–2.11)	0.27 (0.14–0.49)	0.85 (0.74–0.92)	0.56 (0.54–0.56)	0.70
Son ObstetGynecol Sci (2015)	EC patients treated at 1 hospital from 2000–2013	142	3 (2.1%)	Biopsy EEC G1/2	Serum CA125, Biopsy histological grade, MRI MI	4.63 (1.37–6.80)	0.39 (0.07–0.93)	0.67 (0.21–0.94)	0.86 (0.85–0.86)	NA
Sadowski Am J Roentgenol (2015)	EC patients treated at 1 hospital from 2012–2013	99	4 (4.0%)	Biopsy EEC G1/2	Serum CA125, MRI MI, MRI enlarged LNs MRI tumor volume index, MRI cervical invasion	2.97 (1.46–2.97)	0 (0.00–0.74)	1.00 (0.52–1.00)	0.66 (0.64–0.66)	NA

*LNM* Lymph node metastasis, *LH* Likelihood ratio, *CI* Confidence interval, *ROC* Receiver operator characteristic, *NCCH* National Cancer Center Hospital, *SUH* Showa University Hospital, *CA125* Cancer antigen 125, *MRI*Magnetic resonance imaging, *MI* Myometrial.

*Low risk (no risk factor) vs others (> 1 risk factor). **Classified as low risk for LNM in J Clin Oncol (2012).

### Association of the preoperative clinical factors with the risk for LNM between LGEC and HGEC determined by biopsy specimens

In patients with LGEC in the NCCH cohort, multivariate analysis revealed that high serum CA125 levels and enlarged LNs were significantly associated with a risk for LNM (odds ratio [OR] = 7.72, *P* < 0.01, and OR = 9.26, *P* < 0.01, respectively; Table 4A). Multivariate analysis of the SUH cohort showed that high serum CA125 levels were significantly associated with LNM (OR = 13.2, *P* < 0.01; Table 4B). In the combined analysis consisting of 254 patients from the SUH and NCCH cohorts, we identified high serum CA125 levels and enlarged LNs in the LGEC group (OR = 10.1, *P* < 0.01, and OR = 6.20, *P* < 0.01, respectively; Table 4C). Conversely, multivariate analysis revealed that deep MI and high serum CA125 levels in HGEC of the NCCH cohort were significantly associated with the risk of LNM (OR = 9.52, *P* < 0.01, and OR = 17.5, *P* = 0.016, respectively). However, none of the factors were statistically associated with the risk of LNM in the SUH cohort (Table 4A and B). In the combined analysis of 254 patients from the SUH and NCCH cohorts, deep MI on MRI and high serum CA125 levels were associated with the risk of LNM in the HGEC group (OR = 7.86, *P* < 0.01, and OR = 7.32, *P* = 0.019, respectively; Table 4C.

**Table 4:** Association between lymph node metastasis and preoperative clinical risk factors according to the biopsy histological grade.

Category	LGEC						HGEC					
	Univariate			Multivariate			Univariate			Multivariate		
	OR	(95%CI)	*P* value	OR	(95%CI)	*P* value	OR	(95%CI)	*P* value	OR	(95%CI)	*P* value
**(A) NCCH cohort (n = 125, [LGEC: n = 71, HGEC: n = 54])**												
MRI												
Myometrial invasion (≥ 50% / < 50%)	2.81	(1.01–7.80)	0.047	1.79*	(0.50–6.44)	0.38	9.95	(2.85–34.8)	< 0.01	9.52*	(2.37–38.2)	< 0.01
Tumor diameter (high / low)^1^	6.01	(2.06–17.6)	< 0.01	2.18*	(0.58–8.28)	0.25	2.52	(0.84–7.58)	0.10	–	–	–
Enlarged lymph nodes (positive / negative)	10.3	(2.52–41.9)	< 0.01	9.26*	(1.73–49.5)	< 0.01	2.43	(0.62–9.56)	0.20	–	–	–
Serum CA125 level (high / low)^2^	8.74	(2.87–26.6)	< 0.01	7.72*	(2.04–29.2)	< 0.01	18.7	(2.18–160.1)	< 0.01	17.5*	(1.72–178.2)	0.016
**(B) SUH cohort (n = 129, [LGEC: n = 94, HGEC: n = 35])**												
MRI												
Myometrial invasion (≥ 50% / < 50%)	3.12	(1.14–8.52)	0.026	1.99*	(0.62–6.31)	0.24	8.57	(1.39–52.7)	0.021	4.86*	(0.63–37.5)	0.13
Tumor diameter (high / low)^1^	2.61	(0.96–7.09)	0.060	–	–	–	2.43	(0.48–12.3)	0.29	–	–	–
Enlarged lymph nodes (positive / negative)	1.82	(0.31–10.7)	0.51	–	–	–	15.6	(1.34–182.1)	0.028	3.43*	(0.14–82.8)	0.45
Serum CA125 level (high / low)^2^	15.1	(4.46–51.4)	< 0.01	13.2*	(3.83–45.7)	< 0.01	13.3	(2.06–86.3)	< 0.01	4.38*	(0.37–52.1)	0.24
**(C) Combined NCCH cohort and SUH cohort (n = 254)**												
MRI												
Myometrial invasion (≥ 50% / < 50%)	3.14	(1.55–6.38)	< 0.01	1.97**	(0.84–4.63)	0.12	9.37	(3.45–25.5)	< 0.01	7.86**	(2.49–24.8)	< 0.01
Tumor diameter (high / low)^1^	3.86	(1.88–7.93)	< 0.01	1.98**	(0.83–4.70)	0.12	2.51	(1.03–6.09)	0.042	1.18**	(0.36–3.88)	0.79
Enlarged lymph nodes (positive / negative)	6.06	(2.24–16.4)	< 0.01	6.20**	(1.71–22.5)	< 0.01	4.43	(1.36–14.4)	0.013	2.14**	(0.45–10.2)	0.34
Serum CA125 level (high / low)^2^	10.2	(4.69–22.4)	< 0.01	10.1**	(4.11–25.0)	< 0.01	10.8	(3.18–36.9)	< 0.01	7.32**	(1.40–38.3)	0.019

*LGEC* Low-grade endometrial cancer, *HGEC* High-grade endometrial cancer, *OR* Odds ratio, *CI* Confidence interval, *NCCH* National Cancer Center Hospital, *MRI* Magnetic resonance imaging, *CA125* Cancer antigen 125, *SUH* Showa University Hospital.

*Adjusted by statistically significant variables in univariate analysis. **Adjusted by cohort and statistically significant variables in univariate analysis.

^1^High level, > 47 mm. ^2^High level, > 52.3U/mL(unmenopause), > 48U/mL(menopause).

**Figure 2:**
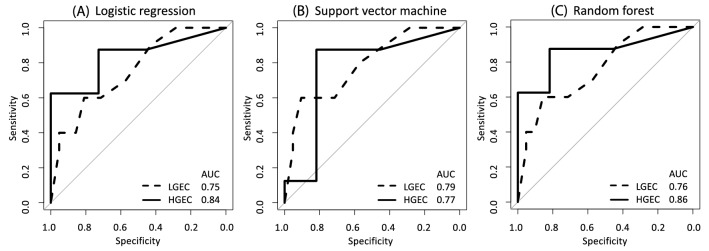
Predictive performance of lymph node metastasis by biopsy histological types (low-grade endometrial cancer / high-grade endometrial cancer (training set: National Cancer Center Hospital (NCCH) training set (*n* = 75), test set: NCCH test set (*n* = 50) and Showa University Hospital (SUH) set (*n* = 129). (**A**) Receiver operating characteristic (ROC) curves using logistic regression. (**B**) ROC curves using support vector machine. (**C**) ROC curves using random forest. Solid and dashed lines show the HGEC and the LGEC test sets, respectively. *AUC* The area under the ROC curve, *HGEC* High-grade endometrial cancer, *LGEC* Low-grade endometrial cancer.

Since the strength of factors contributing to LNM differs between LGEC and HGEC, we decided to separate LGEC and HGEC patients and create LNM prediction models for each. In the LGEC group, the three machine learning methods showed a relatively high predictive ability, with an AUC of approximately 0.75 for the (A) LR: AUC 0.75, (B) SVM: AUC 0.79, (C) RF: AUC 0.76; Fig. 2) models. The HGEC group showed an even higher predictive performance: (A) LR: AUC 0.84, (B) SVM: AUC 0.77, and (C) RF: AUC 0.86.

### Correlation between the predictive classification of LNM and the clinical outcomes

**Figure 3:**
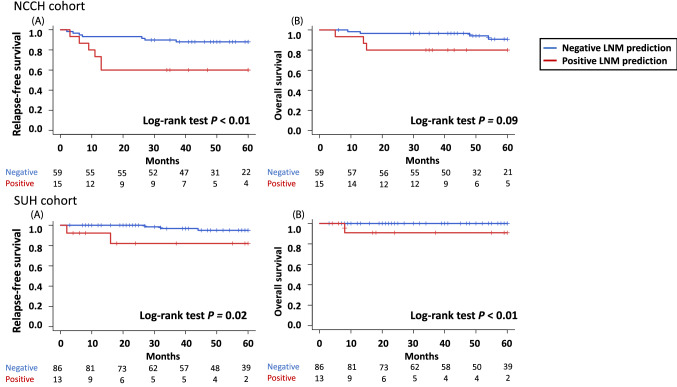
Kaplan–Meier survival curves according to the node-predicted status in patients without pathological lymph node metastasis. Top row, NCCH cohort 125 patients; bottom row, SUH cohort 129 patients. (**A**) RFS of positive lymph node metastasis prediction (red line) and negative lymph node metastasis prediction (blue line). (**B**) OS of positive lymph node metastasis prediction (red line) and negative lymph node metastasis prediction (blue line). *LNM* lymph node metastasis, *NCCH* National Cancer Center Hospital, *OS* Overall survival, *RFS* Relapse-free survival, *SUH* Showa University Hospital.

We examined the association between the LNM predicted by the LR method in this study and the clinical outcomes of 125 patients in the NCCH cohort and 129 patients in the SUH cohort predicted in this study. Patients with positive LNM prediction had better RFS and OS than patients with negative LNM prediction (Supplementary Fig. S1). After adjusting for the presence of adjuvant therapy (chemotherapy or radiation therapy), RFS and OS in the positive LNM prediction group were significantly worse than those in the negative LNM prediction group (Supplementary Table S4). In the group without pathological LNM, the RFS of the groups with positive LNM prediction was worse than that of the groups with negative LNM prediction (Fig. 3). On the other hand, in the group with pathological LNM, there was no significant difference in RFS between the positive and negative LNM prediction groups (Supplementary Fig. S2).

## Discussion

In this study, the LNM prediction model using preoperative clinicopathological predictors showed a high predictive power, similar to that reported previously^19,22–26^. We also showed that the risk factors associated with LNM differ between patients with LGEC and HGEC and that the strength of the association was also different. The prediction performance of the HGEC group was higher than that of the LGEC group. In the group without pathological LNM, patients with positive LNM prediction using our model had worse clinical outcomes than patients with negative LNM prediction, even if 10 or more LNs were removed, and pathology was negative for LNM. These results suggested that this LNM prediction model can identify patients at high risk of recurrence regardless of pathological LNM status, and these patients may require postoperative therapy even if the absence of pathologic LNM.

The predictive model for LNM constructed using clinical factors showed a high AUC of over 0.8, similar to that reported previously^19,22–26^. However, compared to previous reports, the present study resulted in a predictive model with lower sensitivity and higher specificity. The positive likelihood ratio, calculated by sensitivity/ (1—specificity), was higher than or similar to that reported previously. Previous reports have predicted LNM primarily in populations that might have had low LNM risk, which may account for the difference in predictive ability between previous models and the current one. Using the present prediction model, the prediction performance of LNM was better in the HGEC group than in the LGEC group by endometrial biopsy. The group with positive LNM prediction had a poorer prognosis than the group with negative LNM prediction. Due to the high specificity of this model, it could accurately predict poor prognoses of patients who may require lymphadenectomy.

We also showed that LNM prediction using clinical factors had a higher diagnostic performance in the HGEC group than in the LGEC group, and deep MI on MRI correlated with LNM in the HGEC group, and enlarged LNs on MRI correlated with LNM in the LGEC group. In a meta-analysis of the diagnostic precision of clinical biomarkers for the preoperative prediction of LNM in EC, both enlarged LNs detected by MRI and high serum CA125 levels were reported to be more diagnostic of LNM in the HGEC group than in the LGEC group; this is consistent with previous reports^20^.

Clinical factors that are considered to be risk factors for LNM in EC have been reported as poor prognostic factors, and the prediction model for LNM could also possibly predict a population with a poor prognosis. In this study, we revealed, for the first time, that the positive LNM prediction group, including deep MI, large TD, enlarged LN, and high serum CA125 levels, had a worse prognosis, even in patients without postoperative pathological LNM. Many guidelines, including the National Comprehensive Cancer Network, the European Society for Medical Oncology and the Japanese Society of Gynecologic Oncology guidelines, indicate that postoperative pathological stages, histology, and lymph vascular space invasion are parameters for risk assessment in patients with EC^10,32,33^. However, it would be clinically useful to predict prognosis with factors that can be evaluated preoperatively. In the future, the risk of LNM could be calculated based on preoperative pathology information, which could have clinical applications.

Despite its findings, our study had several limitations. This was a two-center, retrospective study with a limited number of patients. The general treatment guidelines for EC patients differed substantially between the two hospitals. This study design might not have included a low-risk metastatic group that did not have their LNs removed. We need to further validate our prediction model with additional independent sample sets because there could be a significant association between LNM risk and HGECs due to differences in histological type distributions by race and surgical methods or treatments administered to high- and low-risk groups for LNM. The previously reported LNM predictive models compared in this study are mostly based on Asian populations and have similar predictive performances. The model used in this study may be useful in predicting poor prognosis patients, particularly in Asian EC patients.

## Conclusion

We demonstrated that routinely assessed preoperative factors can predict LNM with poor prognosis with a high probability independent of the machine learning algorithms used to construct them. The predictive performance of LNM in the HGEC group was as high as AUC 0.84 (as against AUC 0.75 in the LGEC group). Since the clinical factors associated with LNM differ from deep MI and high serum CA125 in the HGEC group to enlarged LNs and high serum CA125 in the LEGC group. The predictive model constructed in this study can also identify patients with a poor prognosis that have aggressive characteristics based on preoperative pathological factors alone, which may provide appropriate treatment selection and surveillance.

## Supplementary Information

Supplementary Information 1.

Supplementary Information 2.

Supplementary Information 3.

## Data Availability

We are unable to upload the clinical data into a public database because we did not receive an agreement from all patients to register their clinical data in a database. However, the authors have described summary data in the paper. Interested researchers may send data requests to Dr. Kouya Shiraishi (kshirais@ncc.go.jp).
