# Supplementary figures and images for: Predictive model for the preoperative assessment and prognostic modeling of lymph node metastasis in endometrial cancer
Source: Sci Rep. 2022 Nov 8;12:19004. doi: 10.1038/s41598-022-23252-3 (PMC9643353; doi:10.1038/s41598-022-23252-3)

Supplementary Figure.S1

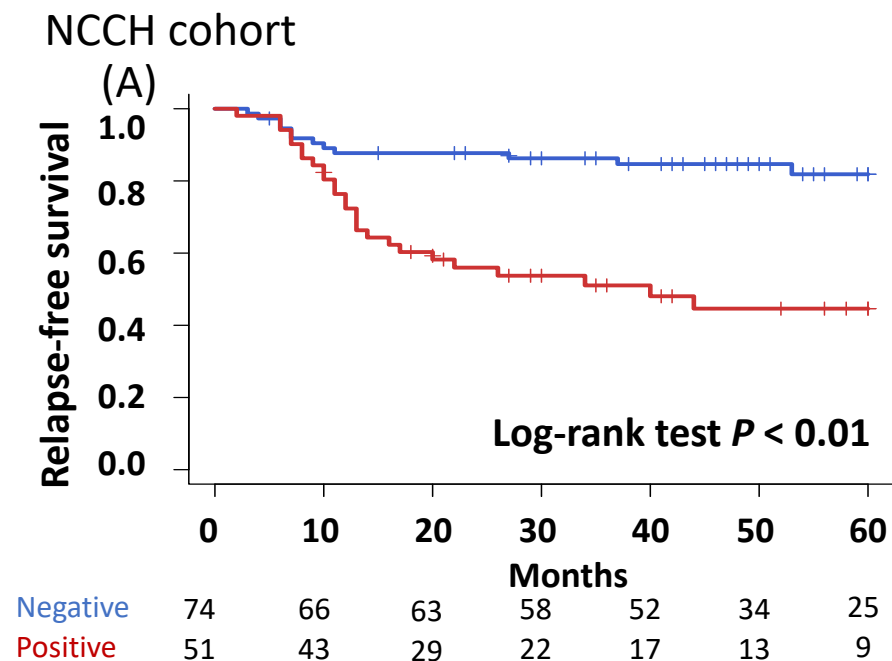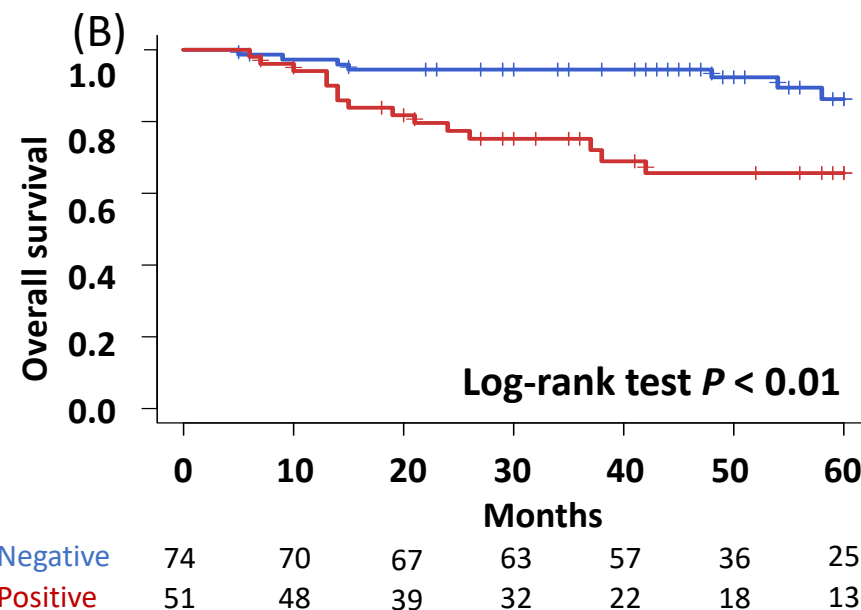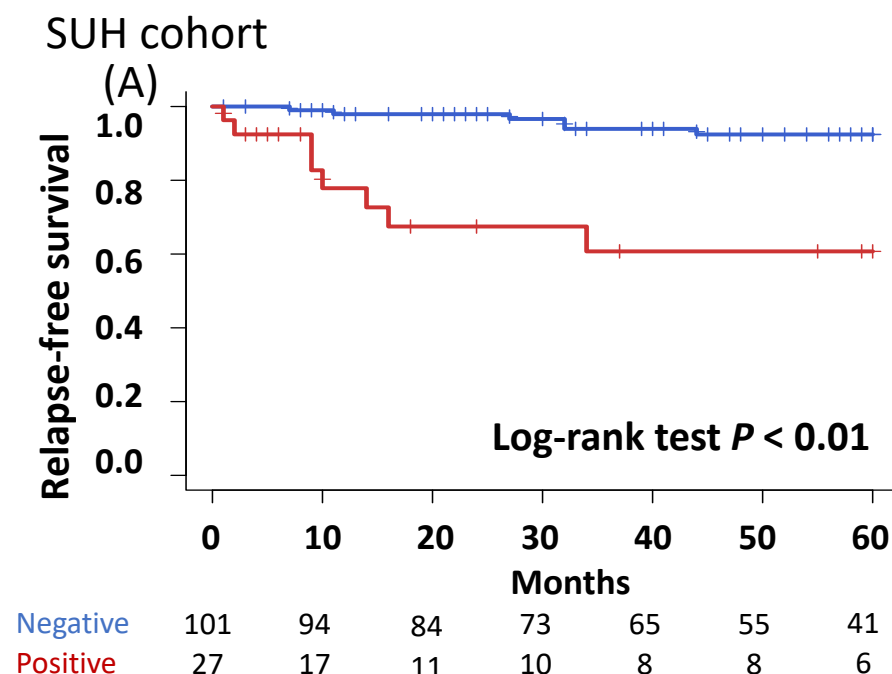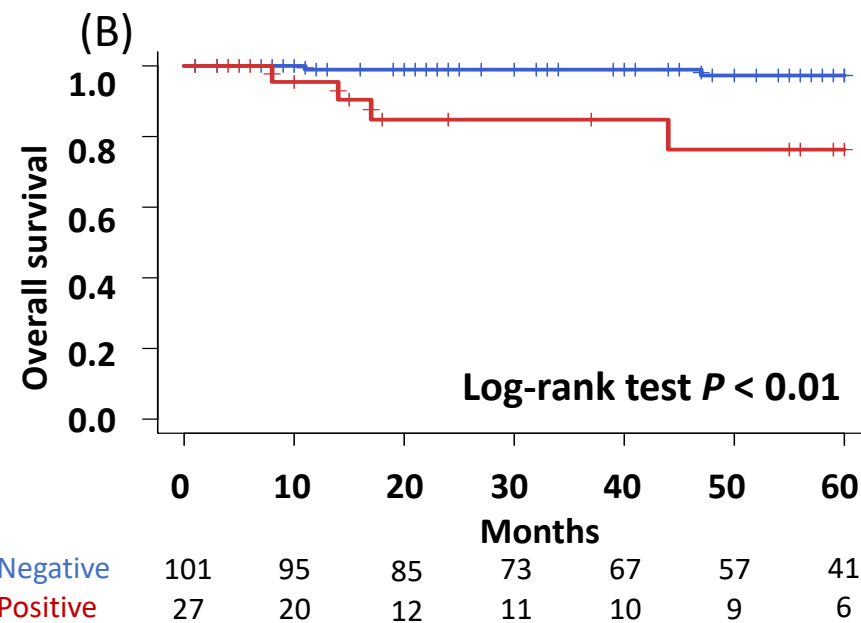

Supplementary Figure.S2

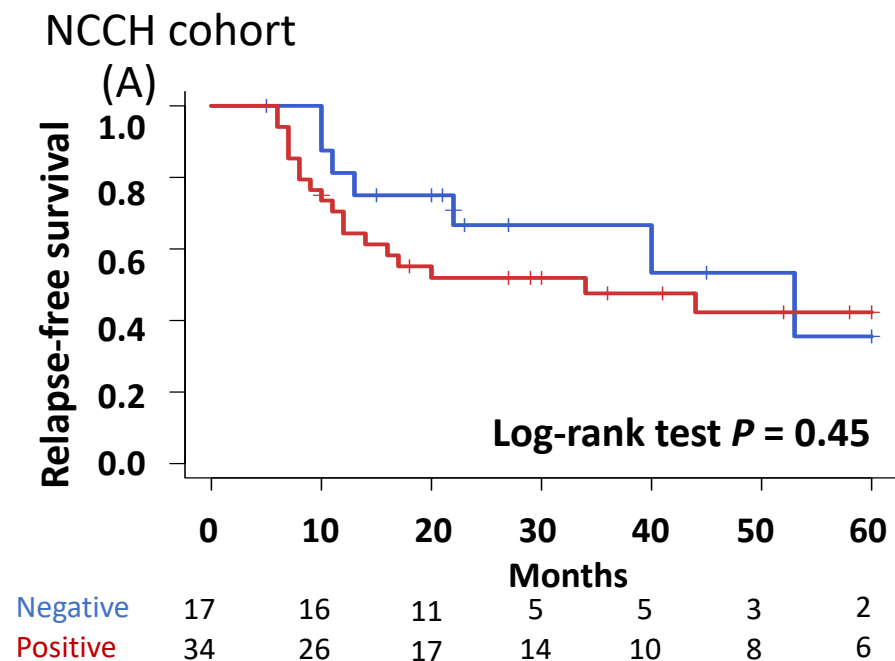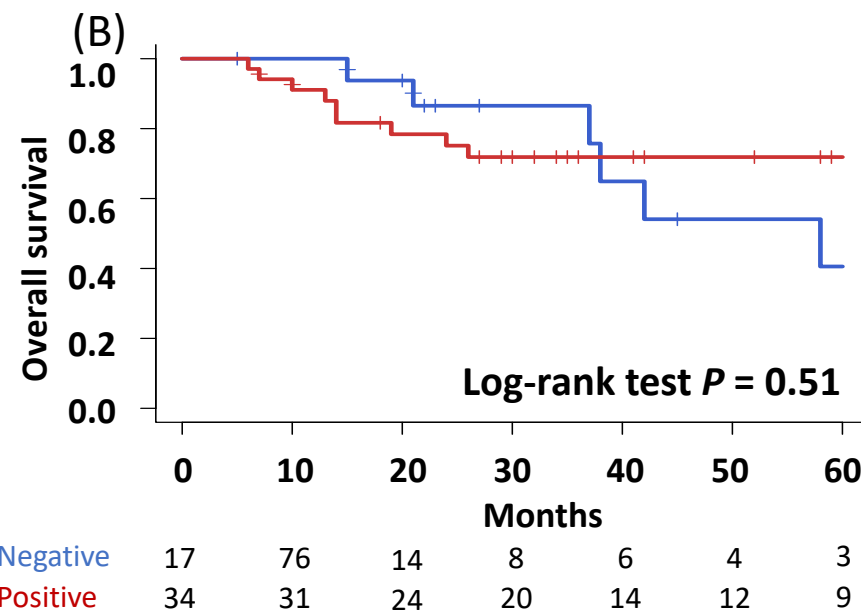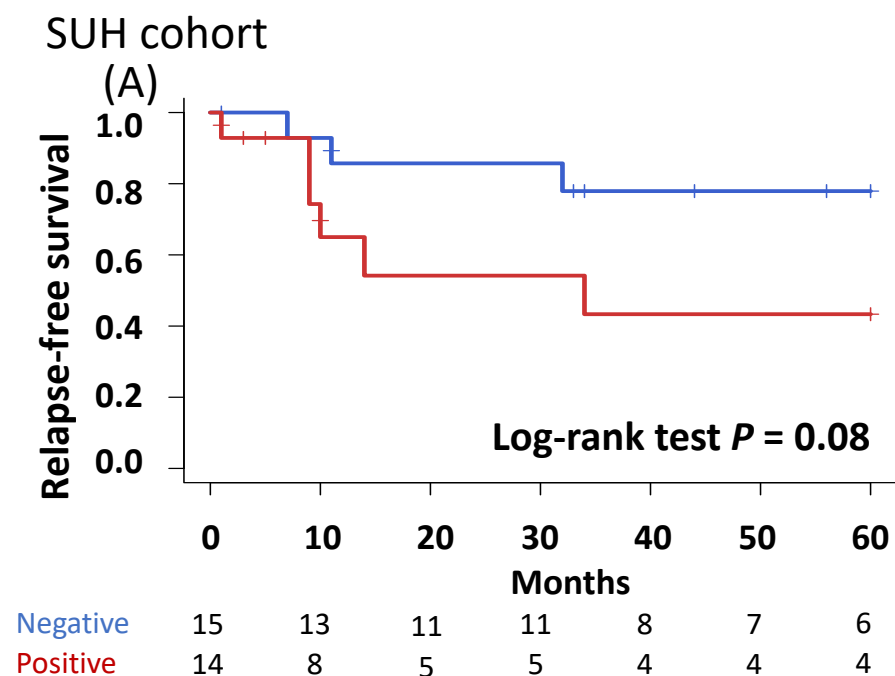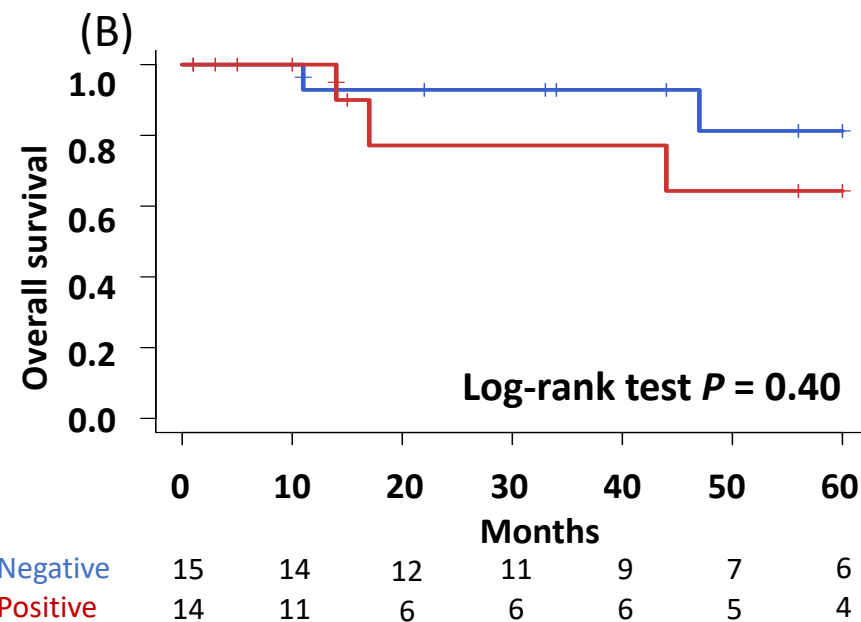

Supplement: Supplementary file 1 — Supplementary Information 1. [file 41598_2022_23252_MOESM1_ESM.pdf]
