# Supplementary material for: Predictive model for the preoperative assessment and prognostic modeling of lymph node metastasis in endometrial cancer
Source: Sci Rep. 2022 Nov 8;12:19004. doi: 10.1038/s41598-022-23252-3 (PMC9643353; doi:10.1038/s41598-022-23252-3)
Supplement: Supplementary file 2 — Supplementary Information 2. [file 41598_2022_23252_MOESM2_ESM.pdf]

Supplementary Material

Predictive model for the preoperative assessment and prognostic modeling of lymph node metastasis in endometrial cancer

Asami Y et al.

Supplementary methods.

Case selections in two hospitals

National Cancer Center Hospital

A total of 840 patients with endometrial cancer underwent surgery between 2007 and 2018. Among them, 151 patients had no lymph node sampled and 345 patients had less than 10 lymph nodes sampled. After excluding 8 patients who did not consent and 211 lacking appropriate medical data for analysis, 125 were enrolled in the study.

Showa University Hospital

A total of 309 patients with endometrial cancer underwent surgery between 2006 and 2017. Among them, 133 patients had no lymph node sampled and 18 patients had less than 10 lymph nodes sampled. After excluding 29 patients due to the lack of appropriate medical data for analysis, 129 patients were enrolled in the study.

Treatment guideline for general endometrial cancer at two hospitals

Preoperatively, we routinely reviewed the treatment strategy for each patient individually with physicians (e.g., gynecologists, radiologists, and oncologists) in both hospitals.

National Cancer Center Hospital

Patients with preoperative diagnosis of stage IA and a low-grade endometrial specimen biopsy underwent intraoperative frozen section diagnosis following lymph node (LN) sampling. If LN metastasis was negative, standard surgery (total hysterectomy and bilateral adnexectomy) was performed; if LN

metastasis was positive, extended surgery (standard surgery and systematic [pelvic and para-aorta LNs] lymphadenectomy) was performed.

Patients with other preoperative diagnosis underwent standard surgery and pelvic lymphadenectomy (PLA), and intraoperative frozen section diagnosis following the removal of multiple LNs was conducted. If LN metastasis was negative, standard surgery and PLA was performed; if LN metastasis was positive, systematic lymphadenectomy was performed.

Showa University Hospital

Patients with low-grade endometrial carcinoma and less than one of half myometrial invasion by intraoperative frozen section diagnosis may be allowed to omit LNs biopsy and dissection, and standard surgery was performed. Other patients underwent extended surgery.

Supplementary Figure legends.

Supplementary Figure S1. Kaplan–Meier survival curves according to LNM prediction status in all patients. Top row, NCCH cohort 125 patients; bottom row, SUH cohort 129 patients. (A) RFS of positive LNM prediction (red line) and negative LNM prediction (blue line). (B) OS of positive LNM prediction (red line) and negative LNM prediction (blue line).

LNM: lymph node metastasis, NCCH: National Cancer Center Hospital, OS: Overall survival, RFS: Relapse-free survival, SUH: Showa University Hospital.

Supplementary Figure S2. Kaplan–Meier survival curves according to the node-predicted status in patients with pathological lymph node metastasis. Upper row, NCCH cohort 125 patients; bottom row, SUH cohort 129 patients. (A) RFS of positive LNM prediction (red line) and negative LNM prediction (blue line). (B) OS of positive LNM prediction (red line) and negative LNM prediction (blue line).

LNM: lymph node metastasis, NCCH: National Cancer Center Hospital, OS: Overall survival, RFS: Relapse-free survival, SUH: Showa University Hospital.
