# Supplementary material for: Predictive model for the preoperative assessment and prognostic modeling of lymph node metastasis in endometrial cancer
Source: Sci Rep. 2022 Nov 8;12:19004. doi: 10.1038/s41598-022-23252-3 (PMC9643353; doi:10.1038/s41598-022-23252-3)
Supplement: Supplementary file 3 — Supplementary Information 3. [file 41598_2022_23252_MOESM3_ESM.pdf]

Supplementary Table S1. Pathological characteristics of resection specimens from 125 patients in the National Cancer Center Hospital cohort.

Category	NCCH cohort	Lymph node metastasis		*P* value
		Positive	Negative	
	[n=125]	[n=51] (43.7%)	[n=74] (56.3%)	
Histological types				0.59**
Endometrioid				
Grade 1	47 (37.6%)	14 (27.5%)	33 (44.5%)	
Grade 2	17 (13.6%)	8 (15.7%)	9 (12.2%)	
Grade 3	18 (14.4%)	8 (15.7%)	10 (13.5%)	
Carcinosarcoma	19 (15.2%)	9 (17.6%)	10 (13.5%)	
Serous	13 (10.4%)	7 (13.7%)	6 (8.1%)	
Mix	5 (4.0%)	2 (3.9%)	3 (4.1%)	
Others	6 (4.8%)	3 (5.9%)	3 (4.1%)	
FIGO stage				<0.01**
IA	35 (28.0%)	0 (0.0%)	35 (47.3%)	
IB	17 (13.6%)	0 (0.0%)	17 (23.0%)	
II	14 (11.2%)	0 (0.0%)	14 (18.9%)	
IIIA	5 (4.0%)	0 (0.0%)	5 (6.8%)	
IIIB	2 (1.6%)	0 (0.0%)	2 (2.7%)	
IIIC1	25 (20.0%)	25 (49.0%)	0 (0.0%)	
IIIC2	16 (12.8%)	16 (31.4%)	0 (0.0%)	
IVB	11 (8.8%)	10 (19.6%)	1 (1.4%)	
Number of resected lymph nodes (median,range)	30 (10-131)	46 (10-131)	21 (10-56)	<0.01*
Extent of lymphadenectomy				<0.01**
Pelvic only	63 (50.4%)	6 (11.8%)	57 (77.0%)	
Pelvic + Para-aortic	62 (49.6%)	45 (88.2%)	17 (23.0%)	
Primary tumor diameter [mm] (median,range)	50 (1.5-184)	55 (1.5-135)	50 (6-184)	<0.01*
Myometrial invasion				<0.01**
<50%	52 (41.6%)	10 (19.6%)	42 (56.8%)	
≥50%	73 (58.4%)	41 (80.4%)	32 (43.2%)	
Lymph vascular space invasion				<0.01**
Negative	51 (40.8%)	5 (9.8%)	46 (62.2%)	
Positive	73 (58.4%)	46 (90.2%)	27 (36.5%)	
Unknown	1 (0.8%)	0 (0.0%)	1 (1.3%)	
Ascites cytology				<0.01**
Negative	79 (63.2%)	22 (43.1%)	57 (77.0%)	
Positive	46 (36.8%)	29 (56.9%)	17 (23.0%)	
Serum CA125 [U/mL] (median, range)	20 (4-1403)	50 (7-1403)	16 (4-448)	<0.01*
Adjuvant therapy				<0.01**
None	66 (52.8%)	3 (5.9%)	63 (85.1%)	
Chemotherapy	58 (46.4%)	47 (92.2%)	11 (14.9%)	
Radiation therapy	1 (0.8%)	1 (2.0%)	0 (0.0%)	
Follow-up period [month] (median, range)	49 (4-137)	29 (5-137)	51 (4-82)	<0.01*

* Mann-Whitney's U test. ** Chi-squared test.

FIGO: The International Federation of Gynecology and Obstetrics, NCCH: National Cancer Center Hospital.

Supplementary Table S2. Pathological characteristics of resection specimens from 129 patients in the Showa University Hospital cohort.

Category	SUH cohort	Lymph node metastasis		*P* value
		Positive	Negative	
	[n=129]	[n=29] (22.5%)	[n=100] (77.5%)	
Histological types				0.14**
Endometrioid				
Grade 1	51 (39.5%)	7 (24.1%)	44 (44.0%)	
Grade 2	37 (28.7%)	9 (31.0%)	28 (28.0%)	
Grade 3	18 (14.0%)	6 (20.7%)	12 (12.0%)	
Carcinosarcoma	6 (4.6%)	2 (6.9%)	4 (4.0%)	
Serous	7 (5.4%)	2 (6.9%)	5 (5.0%)	
Mix	5 (3.9%)	3 (10.4%)	2 (2.0%)	
Others	5 (3.9%)	0 (0.0%)	5 (5.0%)	
FIGO stage				<0.01**
IA	56 (43.4%)	0 (0.0%)	56 (56.0%)	
IB	27 (20.9%)	0 (0.0%)	27 (27.0%)	
II	6 (4.6%)	0 (0.0%)	6 (6.0%)	
IIIA	8 (6.2%)	0 (0.0%)	8 (8.0%)	
IIIB	1 (0.8%)	0 (0.0%)	1 (1.0%)	
IIIC1	8 (6.2%)	8 27.6%)	0 0.0%)	
IIIC2	14 (10.9%)	14 48.3%)	0 0.0%)	
IVB	9 (7.0%)	7 24.1%)	2 2.0%)	
Number of resected lymph nodes (median,range)	49 (13-141)	44.5 (13-121)	60 (14-141)	<0.01*
Extent of lymphadenectomy				<0.01**
Pelvic only	68 (52.7%)	6 (20.7%)	62 (62.0%)	
Pelvic + Para-aortic	61 (47.3%)	23 (79.3%)	38 (38.0%)	
Primary tumor diameter [mm] (median,range)	10.3 (0-88.4)	13.6 (0.45-88.4)	8.8 (0-65.3)	<0.01*
Myometrial invasion				<0.01**
<50%	73 (56.6%)	8 (27.6%)	65 (65.0%)	
≥50%	55 (42.6%)	21 (72.4%)	34 (34.0%)	
Unknown	1 (0.8%)	0 (0.0%)	1 (1.0%)	
Lymph vascular space invasion				<0.01**
Negative	68 (52.7%)	2 (6.9%)	66 (66.0%)	
Positive	50 (38.8%)	22 (75.9%)	28 (28.0%)	
Unknown	11 (8.5%)	5 (17.2%)	6 (6.0%)	
Ascites cytology				0.046**
Negative	91 (70.5%)	17 (58.6%)	74 (74.0%)	
Positive	27 (20.9%)	10 (34.5%)	17 (17.0%)	
Unknown	11 (8.5%)	2 (6.9%)	9 (9.0%)	
Serum CA125 [U/mL] (median, range)	30.5 (5.4-849)	70.3 (13.3-849)	23.2 (5.4-521.3)	<0.01*
Adjuvant therapy				<0.01**
None	60 (46.5%)	5 (17.2%)	55 (55.0%)	
Chemotherapy	67 (51.9%)	24 (82.8%)	43 (43.0%)	
Radiation therapy	2 (1.6%)	0 (0.0%)	2 (2.0%)	
Follow-up period [month] (median, range)	55 (1-124)	50 (1-121)	55 (3-124)	0.91*

* Mann-Whitney's U test. ** Chi-squared test.

FIGO: The International Federation of Gynecology and Obstetrics, , SUH: Showa University Hospital.

Supplementary Table S3. Patient characteristics of the National Cancer Center Hospital cohort: 125 endometrial cancer patients by training or test set.

Category	All [n=125]	Cohort: Training [n=75]	Cohort: Test [n=50]	*P* value
Patients with lymph node metastasis	51 (40.8%)	33 (44.0%)	18 (36.0%)	0.46**
Age, median (range) [years]	60 (29-89)	60 (29-89)	57 (34-82)	0.84*
Histological types by biopsy				0.36**
Low-grade endometrial cancer	71 (56.8%)	40 (53.3%)	31 (62.0%)	
High-grade endometrial cancer	54 (43.2%)	35 (46.7%)	19 (38.0%)	
MRI findings				
Myometrial invasion				0.37**
<50%	61 (48.8%)	34 (45.3%)	27 (54.0%)	
≥50%	64 (51.2%)	41 (54.7%)	23 (46.0%)	
Enlarged lymph nodes				1.00**
Negative	100 (80.0%)	60 (80.0%)	40 (80.0%)	
Positive	25 (20.0%)	15 (20.0%)	10 (20.0%)	
Tumor diameter, median (range) [mm]				
	50 (6-184)	50 (15-120)	50 (6-184)	0.83*
Serum CA125, median (range) [U/mL]	20 (4-1403)	20 (4-1403)	23 (5-270)	0.77*

* Mann-Whitney's U test. ** Chi-squared test.

MRI:Magnetic resonance imaging, CA125: Cancer antigen 125

Supplementary Table S4. Hazard ratios for progression-free survival and overall survival according to prognostic factors in 265 patients with endometrial cancer.

Survival	Cohorts	Variable	Univariate					Multivariate*				
			HR	(	95% CI	)	*P* value	HR	(	95% CI	)	*P* value
Progression free	(A) NCCH cohort (n=125)											
		Presence of adjuvant therapy (presence / absence)	4.05	(	1.96-8.37	)	< 0.01	2.56*	(	1.11-5.88	)	0.03
		Predicted LNM state (positive / negative)	3.91	(	1.96-7.79	)	< 0.01	2.43*	(	1.10-5.36	)	0.03
	(B) SUH cohort (n=129)											
		Presence of adjuvant therapy (presence / absence)	3.36	(	0.94-12.0	)	0.06	1.86*	(	0.48-7.35	)	0.66
		Predicted LNM state (positive / negative)	7.74	(	2.67-22.4	)	< 0.01	6.26*	(	2.01-19.5	)	< 0.01
Overall	(A) NCCH cohort (n=125)											
		Presence of adjuvant therapy (presence / absence)	5.51	(	2.06-14.7	)	< 0.01	3.92*	(	1.30-11.9	)	0.02
		Predicted LNM state (positive / negative)	3.70	(	1.59-8.60	)	< 0.01	1.90*	(	0.74-4.91	)	0.18
	(B) SUH cohort (n=129)											
		Presence of adjuvant therapy (presence / absence)	1.45E+09	(	-	)	0.99	1.09E+9*	(	-	)	0.99
		Predicted LNM state (positive / negative)	10.7	(	1.95-58.8	)	< 0.01	5.84*	(	1.06-32.0	)	0.04

*Adjusted by presence of adjuvant therapy and predicted LNM state.

HR: Hazard ratio. CI: Confidence interval, NCCH: National Cancer Center Hospital, LNM: Lymph node metastasis, SUH: Showa University Hospital.
